# Anaphylactic shock following castor bean contact: a case report

**DOI:** 10.1186/s13223-017-0221-x

**Published:** 2017-11-24

**Authors:** Y. Coattrenec, D. Jaques, P. Jandus, T. Harr, D. Spoerl

**Affiliations:** 1 Division of Immunology and Allergology, Department of Medical Specialties, University Hospital and Medical Faculty, Rue Gabrielle-Perret-Gentil 4, 1211 Geneva, Switzerland; 2 Dr. med. Dominique Jaques, Rue Emile-Yung 11, 1205 Geneva, Switzerland

**Keywords:** Anaphylaxis, Ricin, Allergy, Skin tests, Castor bean

## Abstract

**Background:**

The castor bean plant, *Ricinus communis*, is known to have allergenic and toxic properties. Castor bean allergy has been described mainly as an occupational inhalation allergy in laboratory workers, in persons working in oil processing mills or in agricultural industry. So far, only one case of anaphylactic reaction due to castor bean sensitization confirmed by specific IgE has been described in literature.

**Case presentation:**

A 30-year-old woman presented to the emergency room with severe angioedema followed by urticaria, hypotension and tachycardia. She recovered after treatment with antihistamines, corticosteroids, nebulized adrenaline and intravenous fluids. Food induced anaphylaxis was excluded by allergological investigations. After repeated thorough history, the patient mentioned having bitten into a castor bean just before the reaction. Cutaneous test (prick-to-prick) and specific IgE for castor bean were highly positive.

**Conclusions:**

We report the second case of a severe anaphylactic reaction to castor beans, confirmed by IgE testing, reported in the literature. It underlines the importance of a meticulous history in allergology and highlights the fact, that castor beans may cause potentially fatal anaphylaxis.

## Background

Ricin is a highly toxic protein contained in castor beans that causes inhibition of the ribosomal protein synthesis resulting in cellular damage [1, 2]. When ingested, it mainly causes severe gastroenteritis followed by potentially fatal fluid loss and organ failure. Castor bean allergen-1 (CB1A) is the principal allergen of the castor bean. Whereas ricin is a heat-labile protein, CB1A is a very stable allergen. It consists of low molecular weight albumin storage proteins and is chemically similar to the 2S storage protein of seeds. Ric c1, a 2S storage albumin, was suggested to be one of the major allergens in castor bean and could be detected in most (96%) castor bean-sensitive patients [3]. Castor bean allergens are not only present in the seeds but also in the pollen of this plant [4].

Respiratory symptoms with rhinitis or asthma caused by castor bean dust were reported in several case reports and epidemiological observations [5, 6]. However, only one anaphylactic reaction to castor bean with cutaneous manifestation and circulatory collapse confirmed by specific IgE has been reported up to now [7]. Here, we report the case of a young woman who experienced a severe anaphylactic reaction upon mucosal contact, confirmed by positive skin test and specific IgE for castor bean.

## Case presentation

In November 2015, a 30-year-old woman presented to the emergency department with severe angioedema followed by urticaria, hypotension (systolic pressure 70 mmHg) and tachycardia. Several hours earlier, she had a meal containing meat, mustard, fruits, legumes, cereals, dairy products. Later, she drank beer and vodka. Treatment included nebulized adrenalin, intravenous corticosteroids and fluid resuscitation. She was admitted to the intensive care unit and recovered within 24 h. During the reaction, elevated tryptase levels were documented (61.7 µg/l; standard value < 11.4 µg/l, Thermofisher) that subsequently normalized (5.7 µg/l). Food intake was judged to be unlikely the cause of the anaphylactic reaction, because several hours separated the two events. No causative food or drug could be identified despite extensive allergological investigation including skin tests (vodka, beer) and specific IgE for fruits (hazel nut, brazil nut, orange, apple, cacao, almond, kiwi, melon, banana, grape), legumes (pea, white bean, carrot, potato, tomato, spinach, cabbage, paprika), cereals, egg white, milk, fish, wheat, peanut, soybean, spices (cinnamon, nutmeg, black pepper), sesame seed, yeast, garlic, celery, seafood (fish, shrimp, blue mussel, tuna, salmon), alpha-lactalbumin, house dust mites, dander mix, molds, grass pollen, tree pollen, european ash, and weed pollen as well as oral provocation test for beer and chocolate.

**Fig. 1:**
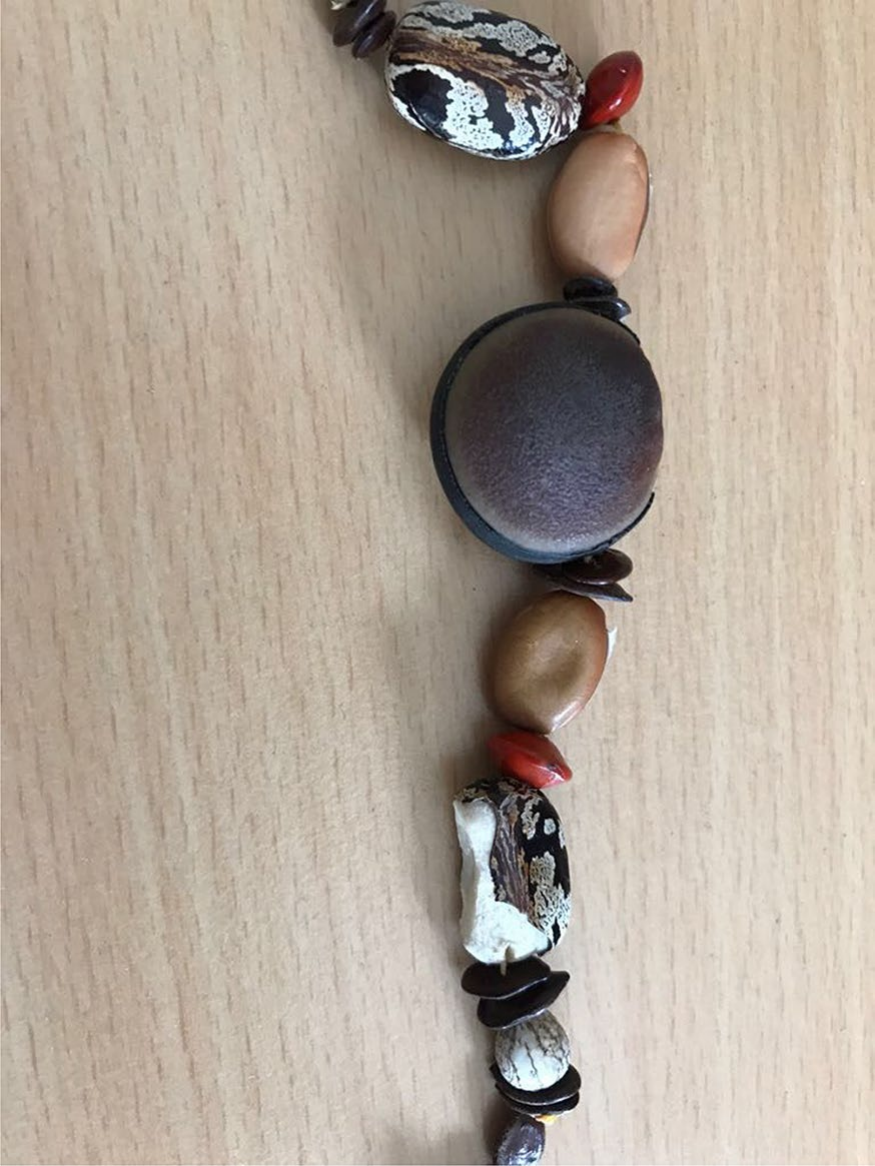
Patient’s necklace containing ricin beans

**Fig. 2:**
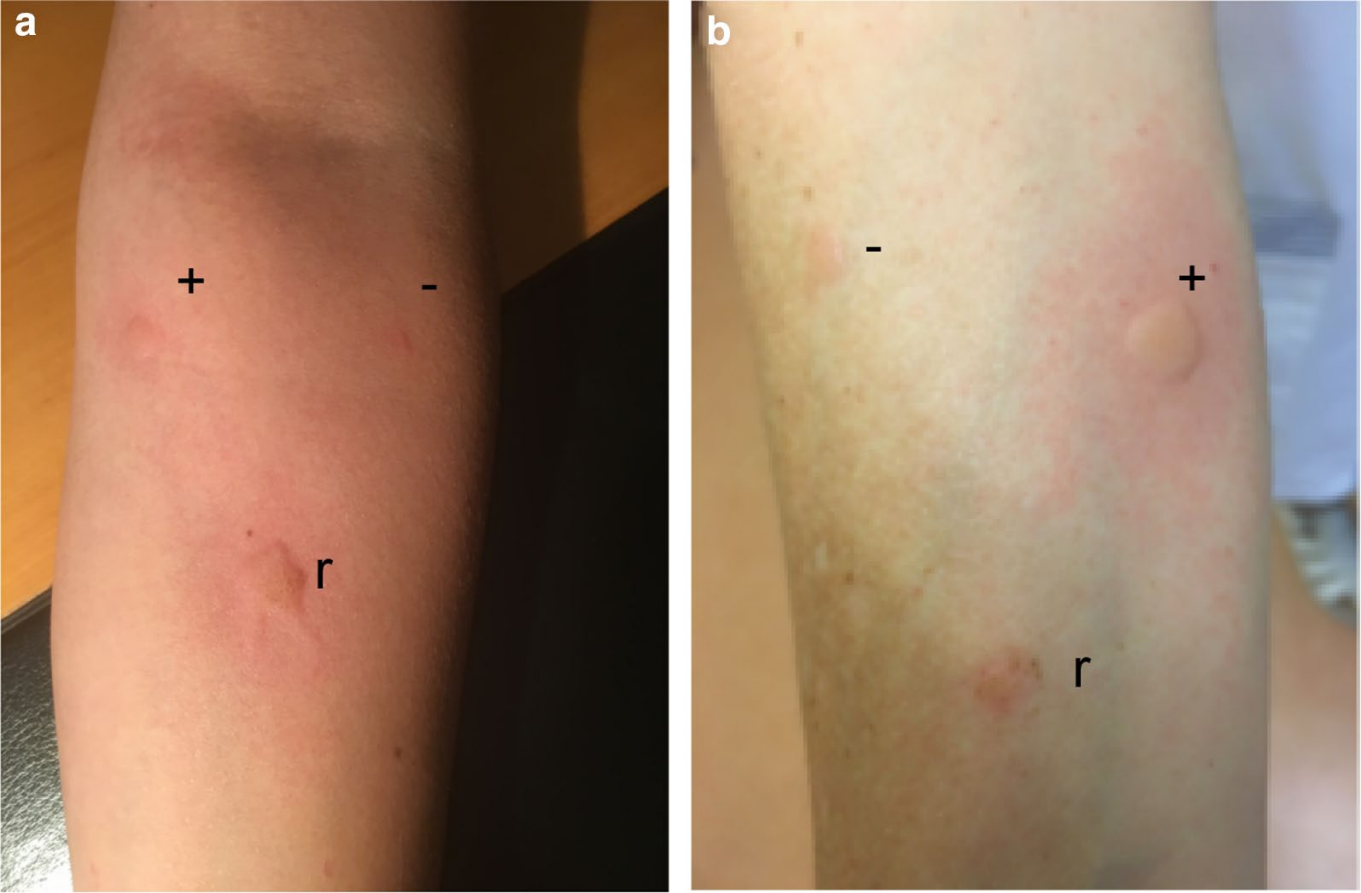
Ricin bean prick-to-prick test for patient (**a**) and healthy control (**b**). +, histamine control; −, negative control; r, ricin

However, after reevaluation, the patient remembered having bitten into a necklace just before the anaphylactic reaction. This necklace was bought in Cuba and consisted of castor beans (Fig. 1). Prick to prick test with this castor bean was strongly positive (Fig. 2) and specific IgE for castor bean were highly positive (91.8 kU/l).

## Discussion and conclusions

*Ricinus communis* is a plant of tropical Africa that grows in warm climates and produces ricin beans. These grains are also called castor beans because they are used in the production of castor oil, which is commercially used as lubricant. Castor beans are used as fertilizer whereas the medical use of the oil is to improve intestinal peristalsis. The bean contains ricin, a highly toxic glycoprotein responsible for severe intoxications. Upon ingestion non-allergic persons generally develop acute gastroenteritis and dehydration. In the most severe cases, hypovolemic shock and possibly fatal organ failure may occur. Intoxication results from the use of castor beans as a purgative or unintentional chewing on beans of a necklace by children. The human lethal oral dose is approximately 1 mg/kg [1].

Castor beans also contain 1.8% of CB1A, which is known to be the principal respiratory allergen. It was first isolated in 1943 [8]. Asthma and rhinitis were reported in South Africa in factory workers of a castor oil company and in people living in the vicinity of the factory [5]. Another outbreak has been reported in the surrounding area of Marseille (France) probably because castor beans were massively imported in this region in later years [6].

Only a few cases of anaphylactic reaction with cutaneous or hemodynamic involvement have been reported in the literature. In 1924, Arnold reported two cases of urticarial rash, edema of the mouth and pharynx following ingestion of a single bean [9]. Recently, the case of woman who developed severe asthma after moving into a new home which was close to castor bean plants has been reported [7]. The same patient developed a severe anaphylactic reaction with angioedema and hypotension after chewing a castor bean seed several years later. She rapidly recovered after treatment with epinephrine, intravenous corticosteroids, antihistamines and intravenous fluids. CAP-RAST to castor bean was 80–100 IU/l [7].

Similarly, our patient suffered from an anaphylactic reaction grade IV (according to Mueller [10]) confirmed by tryptase increase. Specific IgE were highly positive and prick to prick skin test confirmed sensitization to castor bean.

In conclusion, to our knowledge this is the second report of an anaphylactic shock to castor beans confirmed by IgE testing. Mast cell degranulation was confirmed by clearly elevated tryptase levels. Prick to prick test and specific IgE confirmed sensitization to castor bean. This case highlights the importance of obtaining a complete and accurate history of exposures prior to an anaphylactic episode. Castor bean has high allergenic properties and, similarly to peanut storage protein, it may elicit severe anaphylaxis.

## Abbreviations

IgE: immunoglobuline E
CB1A: castor bean allergen-1
CAP-RAST: immunoCAP (serum specific IgE) radioallergosorbent test

## References

[1] Wedin GP, Neal JS, Everson GW, Krenzelok EP (1986). Castor bean poisoning. Am J Emerg Med.

[2] Challoner KR, McCarron MM (1990). Castor bean intoxication. Ann Emerg Med.

[3] Thorpe SC, Kemeny DM, Panzani RC, McGurl B, Lord M (1988). Allergy to castor bean. II. Identification of the major allergens in castor bean seeds. J Allergy Clin Immunol.

[4] Deus-de-Oliveira N, Felix SP, Carrielo-Gama C, Fernandes KV, DaMatta RA, Machado OL (2011). Identification of critical amino acids in the IgE epitopes of Ric c 1 and Ric c 3 and the application of glutamic acid as an IgE blocker. PLoS ONE.

[5] Ordman D (1955). An outbreak of bronchial asthma in South Africa affecting more than 200 persons, caused by castor bean dust from an oil processing factory. Int Arch Allergy Appl Immunol.

[6] Charpin J, Zafiropovlo A (1959). Respiratory allergy to ricin in the Marseille region. Acta Allergol.

[7] Navarro-Rouimi R, Charpin D (1999). Anaphylactic reaction to castor bean seed. Allergy.

[8] Spies JR, Coulson EJ (1943). The chemistry of allergens: isolation and purification of active protein-polysaccharide fraction CB-1A from castor beans. J Am Chem Soc.

[9] Arnold HL (1924). Poisonning from castor beans. Science.

[10] Mueller HL (1966). Diagnosis and treatment of insect sensitivity. J Asthma Res.

